# Supplementary material for: The effect of peer relationships on college students’ behavioral intentions to be physically active: The chain-mediated role of social support and exercise self-efficacy
Source: PLoS One. 2025 May 9;20(5):e0320845. doi: 10.1371/journal.pone.0320845 (PMC12064194; doi:10.1371/journal.pone.0320845)
Supplement: Attachment — Submitted filename: Responses to Editor and Reviewers.doc [file pone.0320845.s002.doc]

**Responses to Editor and Reviewers**

Dear Prof Fan and all the Reviewers,

Happy Chinese New Year. Thank you for your comments concerning our manuscript entitled “*The Effect of Peer Relationships on College Students' Behavioral Intentions to Be Physically Active: the chain-mediated Role of Social Support and Exercise Self-efficacy*”(ID: PONE-D-24-44384).

Thank you for your professional review work, constructive comments, and suggestions on our manuscripts. These comments helped to improve the academic rigor of our articles, and they were beneficial for revising and improving our paper and for highlighting the significance of our research. We have studied the comments carefully and have made corrections that we hope meet with approval.

Based on the comments of the 3 reviewers, we have made corresponding changes, which are marked as highlighted blue in the manuscript for Reviewer 1, light blue for Reviewer 2, and highlighted in yellow for Reviewer 3.

Your sincerely，Zhouxielin; ZhangMu; Chen Lu; Li Bo; Xu Jiao

2025/2/5

Editor#

Additional Editor Comments:

In this manuscript, the authors have made corresponding efforts. The reviewers’ evaluations of the manuscript are not uniform, but I believe the authors should be given another opportunity to revise and improve it. The authors need to carefully address the reviewers’ comments, especially by addressing the theoretical gaps in existing research to elucidate the research value and significance of this study.

Responses to Editor:

We are very grateful for the comments given by the editorial staff, which allowed us to understand the shortcomings of the article and to be able to correct them.

Theoretical support was added to this study. In the introduction, Bandura's Ternary Interaction Theory was used to support why peer relationships, social support, exercise self-efficacy, and physical activity behavioral intention were examined. Bandura’s Ternary Interaction Theory suggests that environmental factors (e.g., peer relationships, social support) form a cyclical dynamic with behavioral intention through personal cognition (e.g., self-efficacy). Based on the ternary interaction theory, this study introduces, peer relations and social support among environmental factors and exercise self-efficacy among personal cognitive factors to explore the influence mechanism of physical activity behavioral intention, aiming at enriching the research results in the existing field, and providing theoretical support for the development of physical education and lifelong physical education in colleges and universities.

In addition, in the first part of the research hypothesis, we added “The theory of planned behavior suggests that an individual's behavior is influenced by his or her behavioral intentions, which in turn are influenced by behavioral attitudes, subjective norms, and perceived behavioral control. Through this theory, peer relationships may influence individuals' physical activity behavioral intentions by influencing their behavioral attitudes and perceptual behavioral control.” To support the proposal of H1.

In the second part of the research hypothesis, we added further attachment theory to support the influence of peer relationships on social support. “On the other hand, John Bowlby's attachment theory emphasizes that individuals seek closeness and attention when forming attachment relationships with others to gain a sense of security and self-confidence, which continues to influence their social behavior and emotion regulation, and from the perspective of attachment theory, the attachment relationships that college students form with their peers during their participation in school-related activities can have an emotions and behaviors in an important way.”

In the third part of the hypothesis, we added Albert Bandura's self-efficacy theory to support the influence of exercise self-efficacy on physical activity behavioral intentions. “Self-efficacy theory (Albert Bandura) suggests that self-efficacy has a significant impact on an individual's behavioral choices, level of motivation, emotional state, and adherence to behavior. Specifically, when individuals have higher self-efficacy, they are more likely to choose challenging tasks and show higher levels of persistence and effort in the face of difficulties.”

In addition, we have further improved the content of the data analysis, the conclusions and recommendations, and the outlook of the study based on the comments of the reviewers.

Finally, I would like to thank again the editorial staff and the reviewers for their suggestions, which enabled us to learn more and improve our research. Happy Chinese New Year to you all.

Reviewer#1:

**Responses to comment: (Revisions are highlighted in blue, for example AAA)**

Q1: However, I think you can delve more into why men outperformed women in exercise self-efficacy and intentions to engage in physical activity. For instance, could this be due to gender roles or societal bias (or other factors) whereby some societies expect men to be more physically fit than women and regard women as weaklings.

A1: We are very grateful to the reviewers for this comment, which helps us to further refine the conclusions of the study and make it more valuable. Based on this issue, we added an analysis of gender. The modifications are as follows：

**Discussion of Gender Differences in Exercise Self-Efficacy and Physical Activity Behavioral Intention**

Compared with other studies, the present study further found, based on previous research, that there were gender differences in exercise self-efficacy and physical activity behavioral intention in the college student population, and that male students scored higher than female students. This difference may stem from the interaction between socio-cultural constructs and individual practical experience. First, gender role theory suggests that gender role expectations (e.g., “being strong” is more often seen as a core element of masculinity) may be influenced through differential socialization processes. That is, males receive early access to athletic resources, whereas females may inhibit the expression of behavioral intentions due to “stereotype threat”. Second, the gradient of accumulated exercise experience exacerbates this difference; in this paper, men had higher exercise self-efficacy than women, and according to Bandura's theory of self-efficacy, the sustained successful experience reinforces the virtuous circle of individual exercise confidence, and thus, men had higher exercise self-efficacy and physical activity behavioral intention than women. Finally, structural environmental factors should not be overlooked. Implicit gender segregation of campus sports spaces (e.g., centralized layout of basketball and soccer courts) may weaken women's sense of belonging to mainstream sports, and thus they face higher psychological costs of participating in physical activity, and their sense of athletic self-efficacy and behavioral intentions to engage in physical activity may be affected. This finding suggests that colleges and universities need to break through the single explanatory framework of physiological differences in their future sports promotion strategies and focus on deconstructing gender barriers in sociocultural constructs, such as developing gender-inclusive physical education and sports curricula and establishing a system of good female role models for sport, to improve the gender differences in self-efficacy for sport and intention to engage in physical activity behaviors.

Details can be found in the revised manuscript on pages 17 to 18, lines 381-404(at the very beginning of the discussion).

Q2: you may need to define the term “good peer relationship” to further contextualize your work. For instance, on number 75 and following numbers, you wrote, 75 when studying issues related to physical activity behaviors [7, 8]. Most studies have found that good 76 peer relationships (?????????????????). It might help to define this concept even if it means putting a synonym in a bracket following it. It is important because this term may be relative. And what is good for a student may be bad for another.

A2: We are very grateful to the reviewers for this comment. Based on the reviewers' comments, we have defined good peer relations to make this concept clearer in this paper. The modifications are as follows：

Some studies have found that good peer relationships (i.e., positive peer interactions through emotional support, joint sports participation, and healthy competition) can promote individuals' engagement in physical activity, which is one of the most important motivations for college students' physical and mental health development. Specifically, under the leadership of peers, individuals will generate behavioral intentions to engage in physical activity through social modeling (e.g., observing peers' exercise behaviors), social facilitation (e.g., peers' presence to enhance exercise performance), and attributional need satisfaction (e.g., integrating into the exercise community), thus participating in sport more actively.

Details can be found in the revised manuscript on page 4, lines 73-80 (Modifications are highlighted in blue).

Q3: However, you can further enhance the study by reporting the effects of age, just as you did for gender. This is important because age can be a determining factor in peer relationship and behavioral intention. For instance, some persons may feel reluctant to associate with people outside their age bracket. So, does age play any role in peer relationship in your study? Also, age may be an influencing factor in students’ behavioral intentions to engage in physical activities. For instance, priorities may change due to age (older students may begin to prioritize academic/work obligations compared to the much younger ones).

A3: We are very grateful to the reviewers for this comment, which has been very beneficial. In the results section of the study, it was obtained that there was no significant difference between peer relationships, social support, exercise self-efficacy, and physical activity behavioral intention in the variable of age, probably because the sample of this study was mainly college students, whose age gap was small. We modified it to read as follows:

Finally, this study found no significant difference in age between peer relationships, social support, exercise self-efficacy, and physical activity behavioral intention, probably because the study group was mainly college students, whose age gap was smaller, and individuals in this age group were closer in physiological and psychological development. The social-ecological system theory emphasizes that individual development is influenced by multi-level environmental factors, including micro-systems (e.g., family, school), meso-systems (e.g., community), and macro-systems (e.g., culture, policy). Based on this theory, the behavior and psychological development of individuals are not only influenced by their own factors, but also by the interaction of the surrounding environment. For college students, they are influenced by similar (schooling, socio-cultural, etc.) environments in the same socio-ecological system, and these similar environmental factors may mask the effects of age on peer relationships, social support, exercise self-efficacy, and physical activity behavioral intentions, resulting in a non-significant age effect.

Details can be found in revised manuscript on page 22 to 23, lines 486-497 (Before the conclusion).

Reviewer#2:

**Responses to comment: (Revisions are in light blue, for example AAA)**

Q1: The sample consists of only 514 students. While this number may be sufficient for certain statistical analyses, it does not adequately capture the diversity of university students, particularly across different cultures or universities with varied educational programs.

A1: We are grateful to the reviewers for this comment, as this study focused on exploring the mechanisms influencing physical activity behavioral intentions in a population of college students, only the demographic variables of gender, school year, and age were considered, and not cross-cultural backgrounds and colleges and universities with different educational systems. In response to this comment, in the section on research subjects, the G*Power test minimum sample size was added to ensure the scientific nature of sample size selection. We modified it to read as follows:

“In this study, before the questionnaire was administered, the minimum sample size was calculated using the G*Power program, setting α=0.05, β=0.80, Effect Size=0.15, to obtain the study's required the minimum sample size was 77”.

In addition, in response to this question from the reviewer, the research team will consider cross-cultural contexts and different educational systems in future studies to explain the relationship more fully.

Details can be found in the revised manuscript on page 10, lines 209-211(This section is in light blue).

Q2：The study is based on theories; however, it does not provide sufficient details on how these theories practically support the hypotheses.

A2: We are very grateful to the reviewers for this comment, which made us realize the lack of support for the theoretical foundation. This study has revised the introduction section based on the suggestion. The revisions are as follows:

Physical activity is an indispensable part of people's lives, as it reflects their sporting values, physical fitness, and mental health. Analyzing from a doctrinal perspective, existing studies have mainly explored the relationships between peer relationships and physical activity behavior, social support and physical activity behavior, and exercise self-efficacy and physical activity behavior. Some studies have found that good peer relationships (i.e., positive peer interactions through emotional support, joint sports participation, and healthy competition) can promote individuals' engagement in physical activity, which is one of the most important motivations for college students' physical and mental health development. Specifically, under the leadership of peers, individuals will generate behavioral intentions to engage in physical activity through social modeling (e.g., observing peers' exercise behaviors), social facilitation (e.g., peers' presence to enhance exercise performance), and attributional need satisfaction (e.g., integrating into the exercise community), thus participating in sport more actively. Although many previous studies have explored the influence mechanism of physical activity and achieved certain results, few scholars have explored the influence mechanism of the antecedent factor of physical activity behavior - the intention of physical activity behavior. So, what is the influence mechanism of physical activity behavior intention? Is it more influenced by intrinsic or extrinsic influences? According to existing research, the formation of the intention to engage in physical activity is a complex psychological process that is influenced by a combination of factors. Bandura's triadic interaction theory suggests that environmental factors (e.g., peer relationships, and social support) form a cyclic dynamic with behavioral intention through personal cognition (e.g., self-efficacy). Based on the ternary interaction theory, this study introduces peer relationship and social support among environmental factors and exercises self-efficacy among personal cognitive factors to explore the influence mechanism of physical activity behavioral intention, aiming at enriching the research results in the existing field and providing theoretical support for the development of physical education and lifelong physical education in colleges and universities.

Details can be found in the revised manuscript on pages 4 to 5, lines 68-92(This section is in light blue).

Q3a: The study did not clarify whether the normality of the data distribution was verified using tests such as Shapiro-Wilk or Kolmogorov-Smirnov, which is essential when employing tests like t-tests or analysis of variance (ANOVA).

A3a: We are very grateful to the reviewers for their comments, which made us aware of the lack of rigor in the previous study. During the revision process, the Kolmogorov-Smirnov test was first performed using SPSS, which showed p<0.05, which may be due to the enhanced statistical efficacy of the test with larger sample sizes, resulting in the possibility of significance of even small deviations from normal distribution. We modified it to read as follows:

According to the central limit theorem, the distribution of sample means can be considered to converge to a normal distribution when the sample size is greater than 200(Armstrong, Covington et al. 2019, Asparouhov and Muthén 2019). The sample size of this study amounted to 514 and the skewness (0.002/0.007) and kurtosis (-1.023/-1.509) of the age and learning strata were significantly lower than the critical values (skewness < 3 and kurtosis < 10) proposed by West et al. (1995), which is in line with the assumption of normality for parametric tests (Dahai 2015).

To support the idea of normal distribution, the prerequisites for conducting t-tests and ANOVA are met.

Details can be found in the revised manuscript on page 14, lines 299-304(This section is in light blue).

Q3b: Regression analysis was used to determine the relationship between variables; however, it was not clarified whether multicollinearity among the independent variables was assessed.

A3b: We are very grateful to the reviewers for their comments. Before the regression analysis, the test of multicollinearity was conducted, and the results of the statistical analysis of covariance, using physical activity behavioral intention as the dependent variable, showed that the VIFs of peer relationship, social support and exercise self-efficacy were 3.381, 1.814 and 3.581, respectively, which were less than 5, and there was no serious multicollinearity, and the results were acceptable. The multicollinearity data are shown in the table below.

| Model |  | Unstandardized coefficient | Standardized coefficient | t | p | covariance statistics |  |
| --- | --- | --- | --- | --- | --- | --- | --- |
|  |  | B | Beta |  |  | tolerances | VIF |
| 1 | (Constant) | .180 |  | 1.659 | .098 |  |  |
|  | peer relationship | .427 | .353 | 7.009 | .000 | .296 | 3.381 |
|  | Social Support | .051 | .047 | 1.278 | .202 | .551 | 1.814 |
|  | Exercise self-efficacy | .475 | .434 | 8.369 | .000 | .279 | 3.581 |
| Dependent variable: physical activity behavioral intentions | | | | | | | |

Details can be found in the revised manuscript on pages 15 to 16, lines 337-342(This section is in light blue).

Q3c: Interaction analysis between independent variables (e.g., social support and peer relationships) was not conducted to determine whether the effects depend on the levels of other variables.

A3c: We are very grateful for the reviewer's comment, which is very helpful for the team's future research. We took your comments very seriously and conducted further analyses. We conducted analyses including interaction effects between social support and peer relationships, social support and exercise self-efficacy, and peer relationships and exercise self-efficacy. The results showed that none of these interaction effects were significant (p > 0.05). In this study, the role of the interaction effect was not considered due to the use of the chain mediation model, where the independent variable was peer relationship, the dependent variable was the intention to engage in physical activity behavior, and the mediating variables were social support and exercise self-efficacy. Although the interaction effect was not significant, the main effect of each variable is still of significant theoretical and practical importance and fulfills the hypotheses of the original study.

Details can be found in the revised manuscript on page 16, lines 342-344(This section is in light blue).

Q4: Although the limitations were mentioned, no clear solutions or future research plans were proposed to address them.

A4: We are very grateful for the reviewers' comments, and based on this comment, the limitations, and specific solutions in the conclusion section have been refined as follows：

Although this study revealed a close relationship between peer relationships, social support, exercise self-efficacy, and physical activity behavioral intentions among college students, there are some shortcomings, and future research can be improved in the following areas:

(1) The fact that this study was cross-sectional and used undergraduate students as the survey population may limit inferences about the causal relationships among the variables. Future research could adopt a longitudinal research design, expand the sample size, and examine the intrinsic mechanisms between the variables in both the longitudinal and cross-sectional dimensions, e.g., undergraduates in different grades could be tracked over time to better understand the trends of these variables over time. In particular, the method of expanding the sample size: although the sample size of this study is 514, which is far above the minimum sample size, in order to further improve the generalizability and reliability of the study, future studies can further expand the sample size to include different regions and types of colleges and universities, for example, stratified sampling by “stage of education”, to compare the differences in mediating effects among different developmental stages, and to examine the mediating effects of different developmental stages. Compare the differences in mediation effects at different stages of development.

(2) While social support and exercise self-efficacy certainly play an important mediating role in exploring the relationship between peer relationships and college students' behavioral intentions to engage in physical activity, there may be other potential mediating variables that play a role in addition. For example, an individual's self-perception, subjective exercise experience, and class belonging. Subsequent studies can be based on this study, starting from the policy context, focusing on the physical and mental health development of adolescents, combining the disciplinary strengths and characteristics of school physical education, and considering the influence of internal and external factors on adolescents' physical exercise behavioral intentions, to provide programs and paths for promoting the comprehensive physical and mental development of adolescents. For example, it is possible to study how factors such as family environment, school policy, and social culture work together to influence the physical activity behavior of college students.

(3) Based on the results of the study, future interventions, such as strengthening mental health education, optimizing the physical education curriculum, and providing more social support, can be designed, and implemented to promote the physical and mental health development of college students. An intervention study can be conducted to observe the effects of measures such as providing psychological counseling and increasing opportunities for physical activity on college students' behavioral intentions to engage in physical activity.

Details can be found in revised manuscript on page 24-25, lines 527-558(This section is in light blue).

Reviewer#3:

**Responses to comment:（Revisions are highlighted in yellow, for example: AAA）**

Q1: The following sentences are lengthy and complex, making comprehension difficult.

"The behavioral intentions to physical activity have a positive impact on an individual's physical and mental health, social development, social adaptation, and study and work, and are also important in an individual's overall development."

A1: We thank the reviewers for their careful comments and have revised the abstract after re-reading it. We modified it to read as follows:

“Behavioral intentions to be physically active have a positive impact on an individual's physical and mental health, social adjustment, and school work.”

The textual content has been highlighted by yellow color.

Details can be found in revised manuscript on page 1, lines 15-16 (This part is highlighted yellow).

Q2: In line 25, what does the author mean by the term ''support''? Does it mean social support?

A2: We are very grateful to the reviewers for their meticulous comments, and after re-reading the abstract, we have revised this section. The revised content is as follows:

The Peer Relationship Inventory, the Physical Activity Rating Scale-Intentions to Physical Activity Behavior subscale, the Social Support Scale, and the Exercise Self-Efficacy Scale were used to psychometrically measure 514 college students (age=19.00±1.27) from June 3, 2024, to June 7, 2024, and the Bootstrap method was used to mediate the relationship between social support and exercise self-efficacy.

Details can be found in revised manuscript on page 1, lines 19-23(This part is highlighted yellow).

Q3: The statement of the problem failed to justify why this study was conducted. The research problem needs major revisions; it must address the novelty of this study and why this study was conducted.

A3: We are very grateful to the reviewers for their comments. Based on the reviewers' comments, we have revised and rewritten the introductory section of the article to highlight the innovative nature of the study. The revisions are as follows:

Behavioral intention to engage in physical activity is an antecedent to individual participation in physical activity and is an important factor affecting the overall development of college students' body and mind. In the face of a complex social environment and severe employment pressure, how to promote their physical and mental health development has become a key concern of the state. However, at present, some students' health awareness is weak, irregular work and rest and many health-damaging lifestyles have become hidden dangers that threaten students' health. According to the survey data of China Youth Net Campus News Agency, the proportion of college students who exercise less than three times a week is as high as 48.19%, and 58.7% of college students exercise for less than 30 minutes each time. The Opinions on Comprehensively Strengthening and Improving School Physical Education Work in the New Era states, “Strengthening the positive role of school physical education work in the new era in promoting students' quality education and all-round development”; and the Special Action Plan for Comprehensively Strengthening and Improving Students' Mental Health Work in the New Era, jointly issued by the Ministry of Education and other seventeen departments, proposes that. “Adhere to the concept of health-first education, effectively place mental health work in a more prominent position and promote the coordinated development of students' ideological and moral qualities, scientific and cultural qualities, and physical and mental health qualities.” The focus of physical education in China has gradually shifted to the development of student's physical and mental health, and the active promotion of college students' commitment to physical exercise and the development of lifelong sports habits has become the core work and important issue in the field of physical education in schools in China.

Physical activity is an indispensable part of people's lives, as it reflects their sporting values, physical fitness, and mental health. Analyzing from a doctrinal perspective, existing studies have mainly explored the relationships between peer relationships and physical activity behavior, social support and physical activity behavior, and exercise self-efficacy and physical activity behavior. Some studies have found that good peer relationships (i.e., positive peer interactions through emotional support, joint sports participation, and healthy competition) can promote individuals' engagement in physical activity, which is one of the most important motivations for college students' physical and mental health development. Specifically, under the leadership of peers, individuals will generate behavioral intentions to engage in physical activity through social modeling (e.g., observing peers' exercise behaviors), social facilitation (e.g., peers' presence to enhance exercise performance), and attributional need satisfaction (e.g., integrating into the exercise community), thus participating in sport more actively. Although many previous studies have explored the influence mechanism of physical activity and achieved certain results, few scholars have explored the influence mechanism of the antecedent factor of physical activity behavior - the intention of physical activity behavior. So, what is the influence mechanism of physical activity behavior intention? Is it more influenced by intrinsic or extrinsic influences? According to existing research, the formation of the intention to engage in physical activity is a complex psychological process that is influenced by a combination of factors. Bandura's triadic interaction theory suggests that environmental factors (e.g., peer relationships, and social support) form a cyclic dynamic with behavioral intention through personal cognition (e.g., self-efficacy). Based on the ternary interaction theory, this study introduces peer relationship and social support among environmental factors and exercises self-efficacy among personal cognitive factors to explore the influence mechanism of physical activity behavioral intention, aiming at enriching the research results in the existing field and providing theoretical support for the development of physical education and lifelong physical education in colleges and universities.

Details can be found in the revised manuscript on pages 3-5, lines 48-93(This part is highlighted yellow).

Q4: The introduction is too long. Why should H1 be examined in the current study if it has already been discussed in previous studies?

A4: We are very grateful to the reviewers for their comments. Most of the previous studies discussed the relationship between peer relationships and physical activity, but there are not many studies on the influence of peer relationships on physical activity behavioral intention, to further explore the extent to which peer relationships influence physical activity behaviors in a group of college students, the present study developed a chain mediation model based on the ternary interaction theory and the theory of planned behavior. Based on the reviewers' suggestions, we revised the first part of the research hypothesis. The revisions are as follows:

Peer relationships are defined as interpersonal relationships between people of the same age or similar levels of psychological development who engage in common activities or collaborate. These relationships are considered to be an important influence on the socialization of individuals as they grow[14]. The establishment of positive peer relationships has been demonstrated to provide university students with the necessary emotional support, thereby reducing the stress of academic and professional obligations. Furthermore, such relationships can also promote participation in individual sports[15]. The notion of behavioral intention to physical activity can be defined as an antecedent to an individual's participation in said activity. This concept refers to an individual's inclination or intention to intentionally engage in physical activity, reflecting an individual's positive attitude and readiness to engage in physical activity. The influence of this concept is derived from a variety of aspects, including personal motivation, the exercise environment, and peer relationships[16-18]. It has been suggested that peer relationships are an important predictor of physical activity behavior[19]. At the same time, in college physical education and health courses, students further improve their cooperation ability through mutual collaboration in course activities and establishing good peer relationships, which can make them more willing to participate in physical exercise. Thus, peer relationships have an important impact on college students' physical fitness and health. In the current research, most of the studies focus on the influence of peer relationships and physical activity behavior, but there are fewer analyses and quantitative studies on the intrinsic mechanism of the intention of peer relationships and physical activity behavior. The Theory of Planned Behavior suggests that an individual's behavior is influenced by his or her behavioral intentions, which in turn are influenced by behavioral attitudes, subjective norms, and perceived behavioral control[20]. Through this theory, peer relationships may influence individuals' behavioral attitudes and perceptual behavioral control by influencing their behavioral intentions, so what is the effect of peer relationships on physical activity behavioral intentions in college student groups? Based on this, this study proposes Hypothesis H1: Peer relationships can significantly and positively predict physical activity behavioral intention.

Details can be found in the revised manuscript on pages 5-6, lines 97-124(This part is highlighted yellow).

Q5: However, similar studies exist exploring peer influence on physical activity. The novelty must be clearly articulated—what does this study add beyond existing work?

A5: We are very grateful for the reviewer's comments, and based on this comment, we have revised the preface section. Previous studies have mostly explored the relationship between peer relationships and physical activity, but fewer studies have explored the relationship between behavioral intentions to be physically active. The revisions are as follows:

Although many previous studies have explored the influence mechanism of physical activity and achieved certain results, few scholars have explored the influence mechanism of the antecedent factor of physical activity behavior - the intention of physical activity. So, what is the influence mechanism of physical activity behavior intention? Is it more influenced by intrinsic or extrinsic influences? According to existing research, the formation of the intention to engage in physical activity is a complex psychological process that is influenced by a combination of factors. Bandura's triadic interaction theory suggests that environmental factors (e.g., peer relationships, and social support) form a cyclic dynamic with behavioral intention through personal cognition (e.g., self-efficacy). Based on the ternary interaction theory, this study introduces peer relationship and social support among environmental factors and exercises self-efficacy among personal cognitive factors to explore the influence mechanism of physical activity behavioral intention, aiming at enriching the research results in the existing field and providing theoretical support for the development of physical education and lifelong physical education in colleges and universities.

Details can be found in the revised manuscript on pages 4-5, lines 80-93(This part is highlighted yellow).

Q6: No control for confounders—e.g., socioeconomic status, prior sports experience, or personality traits, which may also influence exercise self-efficacy.

A6: We are very grateful to the reviewers for their comments, and this study aimed to explore the effects of peer relationships on behavioral intentions to be physically active and to analyze the mediating roles of social support and exercise self-efficacy. Based on the reviewers' suggestions, we recognize that variables such as socioeconomic status, exercise experience, and personality traits are still important, and thus we will further consider these factors in future studies to explore the mechanistic role of physical activity behavioral intentions more fully. Many thanks to the reviewers for their comments.

Details can be found in the revised manuscript on page 25, lines 553-554 (This part is highlighted yellow).

Q7: Ambiguities in Results Presentation:

(1) It is not clear from which scale or questionnaire mobile phone addiction was derived.

(2) In Table 3 (Correlation Coefficients), the sentence "mobile phone addiction was negatively correlated with physical activity behavior" seems out of context.

(3) Figure 2 refers to "Pathways of mobile phone addiction on physical activity behavior."

A7: (1) We are very grateful to the reviewers for this comment. This issue was addressed in the analysis of each variable. The revisions are as follows:

Peer relationships were positively correlated with social support, exercise self-efficacy, and intention to engage in physical activity behavior (p < 0.01), and social support was positively correlated with exercise self-efficacy and intention to engage in physical activity behavior (p < 0.01), and exercise self-efficacy was positively correlated with intention to engage in physical activity behavior (p < 0.01).

Details can be found in the revised manuscript on pages 13 to 14, lines 291-296(This section is highlighted yellow).

(2) We are very grateful to the reviewers for this comment. This issue was addressed in the analysis of each variable. The modifications are as follows:

Peer relationships were positively correlated with social support, exercise self-efficacy, and intention to engage in physical activity behavior (p < 0.01), and social support was positively correlated with exercise self-efficacy and intention to engage in physical activity behavior (p < 0.01), and exercise self-efficacy was positively correlated with intention to engage in physical activity behavior (p < 0.01).

Details can be found in the revised manuscript on pages 13 to 14, lines 291-296 (This section is highlighted yellow).

(3) We are very grateful to the reviewers for this comment, which allowed us to identify the low-level errors we made in the original article, and we will be more careful to avoid these types of low-level errors in our future research. The modifications are as follows:

Figure 2 Pathways of Peer Relationship Influence on Behavioral Intentions to Engage in Physical Activity”

Details can be found in the revised manuscript on page 17, lines 363-364 (This section is highlighted yellow).

Q8: The discussion and conclusion are very poorly written. No comparisons are made between this study and other studies.

A8: We are very grateful to the reviewers for their comments, based on which we have strengthened the discussion and conclusion sections. First, in the “Discussion of gender differences in exercise self-efficacy and physical activity behavioral intention” section, a comparison with previous studies was added to analyze the gender differences in exercise self-efficacy and physical activity behavioral intention in this study. “Compared with other studies, the present study further found, based on previous research, that there were gender differences in exercise self-efficacy and physical activity behavioral intention in the college student population, and that male students had higher scores than female students. This difference may stem from the interaction between socio-cultural constructs and individual practical experience.” Second, in the section “The direct effect of college students' peer relationships on physical activity behavioral intention is significant”, a comparison with previous studies was included. “Based on the previous findings that peer relationships influence physical activity; it was further found that peer relationships can influence physical activity behavioral intention.” Third, in the section “The mediating role of social support between peer relationships and intention to engage in physical activity behaviors,” it is stated that the present study enriches the findings based on previous research. “The present study also found that physical activity behavior intention, as an antecedent of physical activity behavior, was also influenced by social support, further enriching the existing research.” Fourth, in the section “The mediating role of exercise self-efficacy between peer relationships and physical activity behavioral intention”, the following was added: “Meanwhile, the correlations among peer relationships, exercise self-efficacy and physical activity behavioral intention were further enriched based on the findings of the previous studies.” In this section, while previous studies have examined the mediating role of self-efficacy in the influence of peer relationships on physical activity behaviors, the present study further enriches the findings of previous research by highlighting the value of the study, namely, the mediating role of exercise self-efficacy in the intention of peer relationships on physical activity behaviors. Finally, in the conclusion section, we further improved the conclusion content and enriched the recommendations, research deficiencies, and solutions to make the conclusion section more complete.

Details can be found in the revised manuscript on page 18, lines 383-387; page 19, lines 410-412; page 21, lines 456-457; page 21, lines 461-465 (This section is highlighted yellow).

Q9: Discussion & Practical Implications (Weak to Moderate).

A9: We are very grateful to the reviewers for their comments, based on which the recommendations section in the conclusions section has been refined to make its practice revelation content more meaningful. The revisions are as follows:

Therefore, based on the findings of the study, this paper suggests that in order to strengthen college students' peer relationships and promote physical activity and physical activity behavioral intentions, and then promote their overall physical and mental development, colleges and universities can take the following measures: first, organize team sports activities, such as basketball, soccer and other competitions, to enhance teamwork and cohesion; second, set up sports clubs and interest groups to provide a platform for students to participate in sports together Thirdly, to carry out sports training and lectures to improve students' motor skills and sense of self-efficacy; fourthly, to provide adequate sports facilities and resources to ensure that students have good conditions for exercising; fifthly, to strengthen mental health education to help students establish a correct outlook on life and values; and sixthly, to promote peer support, encouraging students to help and encourage each other through trainings and activities.

Details can be found in the revised manuscript on pages 24-25, lines 527-537(This section is highlighted yellow).

Q10: How can universities practically strengthen peer relationships to improve physical activity?

A10: We are very grateful to the reviewers for their comments, based on which the recommendations section in the conclusions section has been refined to make its practice revelation content more meaningful. The revisions are as follows:

Therefore, based on the findings of the study, this paper suggests that in order to strengthen college students' peer relationships and promote physical activity and physical activity behavioral intentions, and then promote their overall physical and mental development, colleges and universities can take the following measures: first, organize team sports activities, such as basketball, soccer and other competitions, to enhance teamwork and cohesion; second, set up sports clubs and interest groups to provide a platform for students to participate in sports together Thirdly, to carry out sports training and lectures to improve students' motor skills and sense of self-efficacy; fourthly, to provide adequate sports facilities and resources to ensure that students have good conditions for exercising; fifthly, to strengthen mental health education to help students establish a correct outlook on life and values; and sixthly, to promote peer support, encouraging students to help and encourage each other through trainings and activities.

Details can be found in the revised manuscript on pages 24-25, lines 527-537(This section is highlighted yellow).

Q11: Are there specific intervention programs that could be developed based on the findings?

A11: We are very grateful to the reviewers for their comments, based on which the specific solutions in the conclusion section were refined. Based on the results of the study, future interventions, such as strengthening mental health education, optimizing physical education curriculum, and providing more social support, can be designed, and implemented to promote the physical and mental health development of college students. An intervention study can be conducted to observe the effects on college students' behavioral intention to be physically active by providing psychological counseling and increasing opportunities for physical activity. The modifications are as follows:

Based on the results of the study, future interventions, such as strengthening mental health education, optimizing the physical education curriculum, and providing more social support, can be designed, and implemented to promote the physical and mental health development of college students. An intervention study can be conducted to observe the effects of measures such as providing psychological counseling and increasing opportunities for physical activity on college students' behavioral intentions to engage in physical activity.

Details can be found in the revised manuscript on pages 26, lines 566-571(This section is highlighted yellow).
